# Dietary calcium, phosphorus, and potassium intake associated with erectile dysfunction in the National Health and Nutrition Examination Survey (NHANES) 2001 to 2004

**DOI:** 10.1371/journal.pone.0297129

**Published:** 2024-02-21

**Authors:** Chen-Yuan Deng, Xin-Peng Ke, Xu-Guang Guo

**Affiliations:** 1 Department of Clinical Laboratory Medicine, Guangdong Provincial Key Laboratory of Major Obstetric Diseases, Guangdong Provincial Clinical Research Center for Obstetrics and Gynecology, The Third Affiliated Hospital of Guangzhou Medical University, Guangzhou, China; 2 Department of Clinical Medicine, The Third Clinical School of Guangzhou Medical University, Guangzhou, China; Tehran University of Medical Sciences, ISLAMIC REPUBLIC OF IRAN

## Abstract

**Background:**

Erectile dysfunction is now a common disorder of sexual function, and its relationship to dietary calcium, phosphorus, and potassium has not been well studied. We set out to determine if dietary intakes of calcium, phosphorus, and potassium are related to erectile dysfunction in U.S. men.

**Methods:**

For this cross-sectional investigation, we used data from NHANES 2001–2004. To investigate the connection of dietary calcium, phosphorus, and potassium intake with erectile dysfunction, we employed multivariate logistic regression, smoothed curve fitting, and subgroup analysis.

**Results:**

This cross-sectional study comprised 3,556 eligible male subjects in total, with a weighted mean age of 49.93±18.13 years. After controlling for race and age, the greatest tertile of calcium consumption was found to have a 34% lower risk of erectile dysfunction than the lowest tertile (OR = 0.66; 95% CI = 0.52–0.84; *p* = 0.0006). The risk of erectile dysfunction was found to be reduced by 33% (OR = 0.67; 95% CI = 0.52–0.87; *p* = 0.0024) for the highest tertile of phosphorus intake compared to the lowest tertile of phosphorus intake and by 35% (OR = 0.65; 95% CI = 0.50–0.83; *p* = 0.0006) for the highest tertile of potassium intake compared to the lowest tertile of potassium intake in the fully adjusted model.

**Conclusion:**

Erectile dysfunction and dietary consumption of calcium, phosphorus, and potassium are inversely associated with the U.S. population. To confirm the accuracy of our findings, additional prospective studies are necessary. Furthermore, it is imperative to do further fundamental research at the molecular level to gain a deeper understanding of the underlying mechanisms.

## 1. Introduction

One of the most prevalent illnesses affecting men is erectile dysfunction, which is both an organic and a psychological condition. Couples’ quality of life is affected, in addition to the patient’s emotional difficulties [1]. The inability to obtain or maintain an erection strong enough for satisfying sexual performance is known as erectile dysfunction [2]. Approximately 180,000 males in the United States today experience erectile dysfunction, according to statistics that are not complete [3]. There is mounting evidence that erectile dysfunction is developing at younger ages [4]. Diabetes and cardiovascular disease are two main risk factors for erectile dysfunction. Endothelial dysfunction, inflammation, and low plasma testosterone levels are all pathophysiological characteristics that are shared by erectile dysfunction and cardiovascular disease. Erectile dysfunction is regarded as an early sign of subclinical vascular disease [5,6].

Common nutrients like calcium, phosphorus, and potassium are included in almost all of our daily meals. Increased potassium intake lowers the risk of cardiovascular disease as well as the incidence of stroke, demonstrating the strong connection between dietary potassium and cardiovascular health [7]. The impact of supplemental calcium and phosphorus on cardiovascular outcomes, however, is still debatable [8,9]. Therefore, further research is needed to determine how calcium, phosphorus, and potassium intake affect erectile dysfunction.

This cross-sectional study investigated the associations of calcium, phosphorus, and potassium intake with erectile dysfunction using information from the National Health and Nutrition Examination Survey (NHANES) conducted between 2001 and 2004. We predicted that associations of calcium, phosphorus, and potassium intake with erectile dysfunction would be detrimental.

## 2. Methods

### 2.1 Sources of data and samples

All data in this analysis were obtained from NHANES, a cross-sectional survey of the U.S. population conducted on a 2-year cycle by the National Centre for Health Statistics (NCHS). This survey collects information for the study of a variety of diseases and elements of life health. And our study used a stratified multistage probability sampling procedure, so the sample included was well represented [10]. The NCHS Ethics Review Board cleared human subjects for NHANES, and all participants provided signed informed consent.

**Fig 1:**
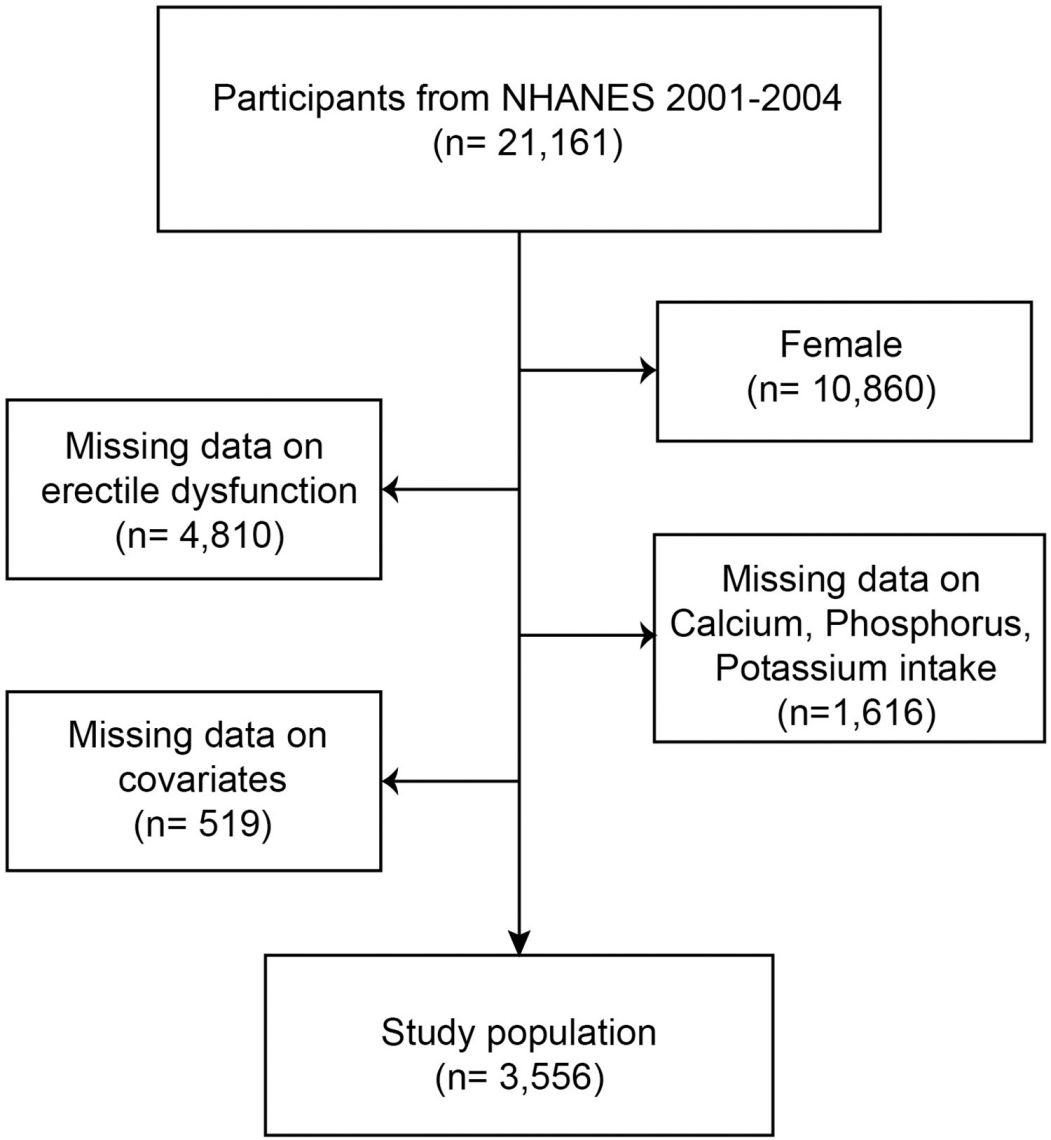
Flowchart of the sample selection from NHANES 2001–2004. NHANES: National Health and Nutrition Examination Survey.

Data from the five NHANES cycles 2001–2004 were selected for this investigation because complete data on calcium, phosphorus, potassium intake, and erectile dysfunction were only available for these two cycles, and a total of 21,161 subjects were initially included. Women (n = 10,860), people lacking information on erectile dysfunction (n = 4,810), people lacking information on calcium, phosphorus, and potassium intake (n = 1,616), and people lacking information on covariates (n = 519) were all eliminated. In the end, 3,556 individuals participated in this cross-sectional study (Fig 1).

### 2.2 Exposing variables and ending variable definitions

Calcium, phosphorus, and potassium consumption were classified as exposure variables. Calcium, phosphorus, and potassium intake included dietary supplements as well as total nutrient intake. A total of two 24-hour food recall interviews were open to everyone. A mobile examination center (MEC) conducted the initial dietary recall interview in person. Three to ten days later, the interview was conducted by phone [11].

Erectile dysfunction was the ending variable. The Audio Computer Assisted Self Interview (ACASI) was used to assess erectile dysfunction. "Ability to maintain an erection (KIQ400)." If the individual answered "Always or almost always able" or "Usually able," he was regarded to be erectile dysfunction-free. The subject was diagnosed with erectile dysfunction if the answer was "Sometimes able" or "Never able."

### 2.3 Covariates

Age (years), race (Mexican American/Other Hispanic/Non-Hispanic White/Non-Hispanic Black/Other Race), education level (Less than high school/High school/More than high school), marital status (Married/Living with partners, Widowed/Divorced/Separated, Never married), income-to-poverty ratio (PIR), alcohol use (Yes/No), smoking (Yes/No), physical activity (Yes/No), hypertension (Yes/No), diabetes (Yes/No), body mass index (BMI) (kg/m^2^), total cholesterol (mg/dL), and high-density lipoprotein (HDL) (mg/dL) were all included as covariates in this study to reduce the influence of potential factors on the relationships between exposure variables and outcome variables [12,13]. The complete measurement methods for these variables are all accessible to the public at https://www.cdc.gov/nchs/nhanes/.

### 2.4 Statistical analysis

The Centers for Disease Control and Prevention (CDC) guidelines provided guidance for the statistical analysis of this study. The statistical analyses for this study were guided by the CDC recommendations, and we took into account the weighting of the NHANES sample collection during the analyses. To compare participant differences between those with and without erectile dysfunction, weighted Student’s t-tests for continuous variables and weighted chi-square testing for categorical variables were utilized. In each of the three models, multifactorial logistic regression models were used to investigate the associations of calcium, phosphorus, and potassium consumption with erectile dysfunction. Model 1 is a crude model that does not adjust for any of the covariates. Model 2 adjusts for age and race. And Model 3 adjusts for all covariates that were included in this study, which is a fully adjusted model. Additionally, subgroup studies of the relationships of calcium, phosphorus, and potassium consumption with erectile dysfunction were carried out. R (version 4.1.3) and EmpowerStats (version 2.0) are statistical computations and graphics programs that were used to conduct the study. And *p* < 0.05 was considered statistically significant.

## 3. Results

### 3.1 Baseline characteristics of participants

This study comprised 3,556 eligible patients with a mean age of 49.93±18.13 years, including 2,411 who did not have erectile dysfunction and 945 who did. The weighted mean calcium, phosphorus, and potassium intakes were 925.28±599.15 mg, 1487.04±692.09 mg, and 3017.88 ±1336.68 mg, respectively. Age, race, education level, marital status, PIR, physical activity, alcohol use, smoking, hypertension, diabetes, BMI, total cholesterol, and high-density lipoprotein all showed statistically significant differences among those with and without erectile dysfunction (all *p*<0.05) (Table 1).

**Table 1:** Baseline characteristics of the study population based on the presence or absence of erectile dysfunction.

	Total (n = 3 556)	No ED (n = 2 411)	ED (n = 945)	*P*-value
**Age (years)**	49.93 ± 18.13	43.78 ± 15.39	65.62 ± 14.86	<0.001
**PIR**	2.86 ± 1.60	2.95 ± 1.61	2.62 ± 1.54	<0.001
**BMI (kg/m^2)**	28.08 ± 5.42	27.91 ± 5.28	28.51 ± 5.75	0.012
**Total cholesterol (mg/dL)**	200.47 ± 44.95	201.71 ± 44.64	197.31 ± 45.62	0.002
**High-density lipoprotein (mg/dL)**	47.44 ± 12.99	47.42 ± 13.01	47.50 ± 12.96	0.825
**Calcium intake (mg)**	925.28 ± 599.15	977.68 ± 631.86	791.60 ± 481.28	<0.001
**Phosphorus intake (mg)**	1487.04 ± 692.09	1568.44 ± 727.93	1279.36 ± 538.02	<0.001
**Potassium intake (mg)**	3017.88 ± 1336.68	3135.29 ± 1394.35	2718.32 ± 1123.17	<0.001
**Race**				<0.001
Mexican American	666 (19.85%)	485 (20.12%)	181 (19.15%)	
Other Hispanic	115 (3.43%)	80 (3.32%)	35 (3.70%)	
Non-Hispanic White	1882 (56.08%)	1307 (54.21%)	575 (60.85%)	
Non-Hispanic Black	590 (17.58%)	455 (18.87%)	135 (14.29%)	
Other Race	103 (3.07%)	84 (3.48%)	19 (2.01%)	
**Education level**				<0.001
Less than high school	891 (26.55%)	529 (21.94%)	362 (38.31%)	
High school	834 (24.85%)	632 (26.21%)	202 (21.38%)	
More than high school	1631 (48.60%)	1250 (51.85%)	381 (40.32%)	
**Marital status**				<0.001
Married/Living with partners	2377 (70.83%)	1661 (68.89%)	716 (75.77%)	
Widowed/Divorced/Separated	448 (13.35%)	279 (11.57%)	169 (17.88%)	
Never married	531 (15.82%)	471 (19.54%)	60 (6.35%)	
**Alcohol use**				0.033
Yes	2777 (82.75%)	2016 (83.62%)	761 (80.53%)	
No	579 (17.25%)	395 (16.38%)	184 (19.47%)	
**Hypertension**				<0.001
Yes	1049 (31.26%)	550 (22.81%)	499 (52.80%)	
No	2307 (68.74%)	1861 (77.19%)	446 (47.20%)	
**Diabetes**				<0.001
Yes	345 (10.28%)	124 (5.14%)	221 (23.39%)	
No	3011 (89.72%)	2287 (94.86%)	724 (76.61%)	
**Smoking**				<0.001
Yes	1995 (59.45%)	1332 (55.25%)	663 (70.16%)	
No	1361 (40.55%)	1079 (44.75%)	282 (29.84%)	
**Physical activity**				<0.001
Yes	1980 (61.05%)	1518 (64.00%)	462 (53.04%)	
No	1263 (38.95%)	854 (36.00%)	409 (46.96%)	

PIR, income- to- poverty ratio; BMI, body mass index.

### 3.2 Association of calcium, phosphorus, and potassium consumption with erectile dysfunction

When considered as a continuous variable, the relationships of calcium, phosphorus, and potassium intake with erectile dysfunction are shown in S1 Table. Upon adjusting for all potential confounders, we discovered that while there were statistically significant correlations of calcium (OR = 0.9998; 95% CI = 0.9996–1.0000; *p* = 0.047), phosphorus (OR = 0.9998; 95% CI = 0.9996–0.9999; *p* = 0.005), and potassium intake (OR = 0.9998; 95% CI = 0.9998–0.9999; *p*<0.001) with erectile dysfunction, the effects were weak. Consequently, we went one step further and transformed the continuous variable of calcium, phosphorus, and potassium intake into a categorical variable (tertiles).

The association between tertiles of calcium intake and erectile dysfunction is displayed in Table 2. In the crude model, calcium consumption is found to be inversely correlated with erectile dysfunction, with a 55% reduction in the incidence of erectile dysfunction in the highest tertile of calcium intake compared to the lowest tertile of calcium intake (OR = 0.45; 95% CI = 0.37–0.55; *p*<0.0001). Even in Model 2, this relationship was maintained (OR = 0.66; 95% CI = 0.52–0.84; *p* = 0.0006). But in Model 3, the *p*-trend is more than 0.05.

**Table 2:** Relationship between tertiles of calcium intake and erectile dysfunction.

Exposure	OR (95%CI), *P*-value		
	Model 1	Model 2	Model 3
Calcium intake tertiles			
T1 (23–605 mg)	1.00 (Reference)	1.00 (Reference)	1.00 (Reference)
T2 (605.5–1,021 mg)	0.70 (0.59, 0.84) 0.0001	0.71 (0.57, 0.88) 0.0020	0.83 (0.65, 1.05) 0.1136
T3 (1,022–8,701 mg)	0.45 (0.37, 0.55) <0.0001	0.66 (0.52, 0.84) 0.0006	0.77 (0.60, 0.99) 0.0453
*p*-trend	<0.0001	0.0008	0.0523

Model 1: Crude mode.

Model 2: Adjusted for age and race.

Model 3: Adjusted for age, race, education level, marital status, income-to-poverty ratio (PIR), alcohol use, smoking, physical activity, hypertension, diabetes, body mass index (BMI), total cholesterol, and high-density lipoprotein (HDL).

Table 3 shows the connection between tertiles of phosphorus intake and erectile dysfunction. Phosphorus consumption was related to erectile dysfunction adversely in all three models. In Model 3, the risk of erectile dysfunction was statistically significantly reduced by 33% in the greatest phosphorus intake tertile compared to the lowest phosphorus intake tertile (OR = 0.67; 95% CI = 0.52–0.87; *p* = 0.0024). Between T1 and T2, there was, however, no statistical difference (OR = 0.88; 95% CI = 0.70–1.10; *p* = 0.2657).

**Table 3:** Relationship between tertiles of phosphorus intake and erectile dysfunction.

Exposure	OR (95%CI), *P*-value		
	Model 1	Model 2	Model 3
Phosphorus intake tertiles			
T1 (84–1,137.5 mg)	1.00 (Reference)	1.00 (Reference)	1.00 (Reference)
T2 (1,138–1,647 mg)	0.69 (0.58, 0.82) <0.0001	0.81 (0.65, 1.00) 0.0537	0.88 (0.70, 1.10) 0.2657
T3 (1,648–8,194 mg)	0.33 (0.27, 0.40) <0.0001	0.62 (0.49, 0.79) 0.0001	0.67 (0.52, 0.87) 0.0024
*p*-trend	<0.0001	0.0001	0.0024

Model 1: Crude mode.

Model 2: Adjusted for age and race.

Model 3: Adjusted for age, race, education level, marital status, income-to-poverty ratio (PIR), alcohol use, smoking, physical activity, hypertension, diabetes, body mass index (BMI), total cholesterol, and high-density lipoprotein (HDL).

The correlation between tertiles of potassium intake and erectile dysfunction is shown in Table 4. Potassium intake was changed from a continuous variable to a categorical variable (tertiles) for sensitivity analysis. In fully adjusted models (Model 3), the highest tertile of potassium consumption was found to significantly reduce the risk of having erectile dysfunction by 35% when compared to the lowest tertile of potassium intake (OR = 0.65; 95% CI = 0.50–0.83; *p* = 0.0006).

**Table 4:** Relationship between tertiles of potassium intake and erectile dysfunction.

Exposure	OR (95%CI), *P*-value		
	Model 1	Model 2	Model 3
Potassium intake tertiles			
T1 (183–2,353 mg)	1.00 (Reference)	1.00 (Reference)	1.00 (Reference)
T2 (2,354–3,357 mg)	0.74 (0.62, 0.88) 0.0008	0.78 (0.62, 0.97) 0.0243	0.77 (0.61, 0.98) 0.0332
T3 (3,359–13,969 mg)	0.48 (0.40, 0.58) <0.0001	0.60 (0.47, 0.75) <0.0001	0.65 (0.50, 0.83) 0.0006
*p*-trend	<0.0001	<0.0001	0.0007

Model 1: Crude mode.

Model 2: Adjusted for age and race.

Model 3: Adjusted for age, race, education level, marital status, income-to-poverty ratio (PIR), alcohol use, smoking, physical activity, hypertension, diabetes, body mass index (BMI), total cholesterol, and high-density lipoprotein (HDL).

**Fig 2:**
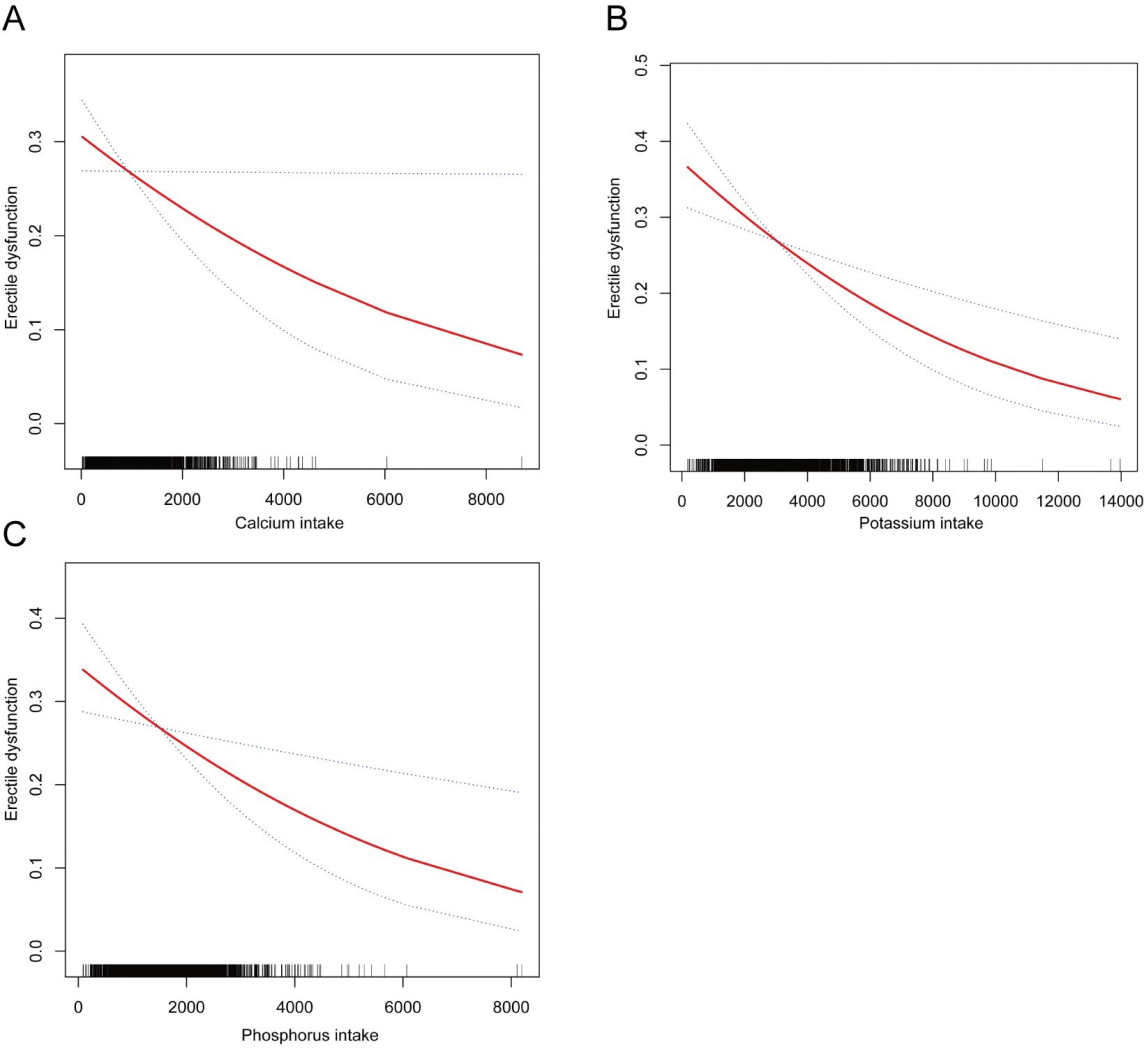
Smoothed curve fitting for calcium, phosphorus, potassium intake, and erectile dysfunction. (A) Smooth curve fitting between calcium intake and erectile dysfunction; (B) Smooth curve fitting between potassium intake and erectile dysfunction; (C) Smooth curve fitting between phosphorus intake and erectile dysfunction.

Additionally, the correlations between calcium, phosphorus, and potassium consumption with erectile dysfunction were visualized using the results of smoothed curve fitting, and we were unable to detect a non-linear association (Fig 2).

### 3.3 Subgroup analysis

We used stratified weighted multivariate regression analyses to conduct subgroup analyses stratified by age, race, BMI, hypertension, and diabetes in order to further investigate the relationships of calcium, phosphorus, and potassium consumption with erectile dysfunction in various populations.

**Fig 3:**
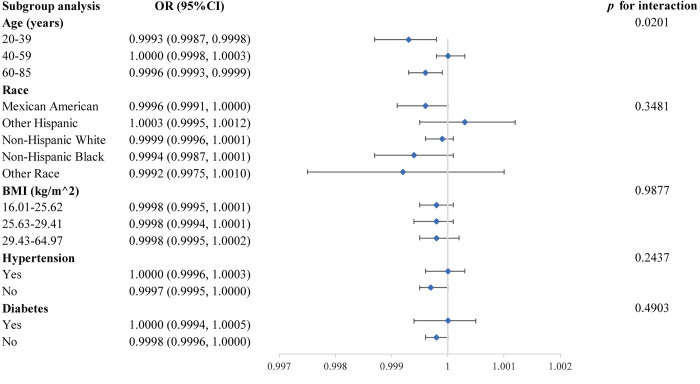
Subgroup analysis for the association between calcium consumption and erectile dysfunction.

Fig 3 depicts a subgroup analysis of the association between calcium consumption and erectile dysfunction, in which we found a significant interaction at age (*p* for interaction<0.05). At the same time, this correlation was unaffected by race, BMI, hypertension, or diabetes, while the negative association between calcium consumption and erectile dysfunction persisted in Mexican Americans with no hypertension or diabetes.

**Fig 4:**
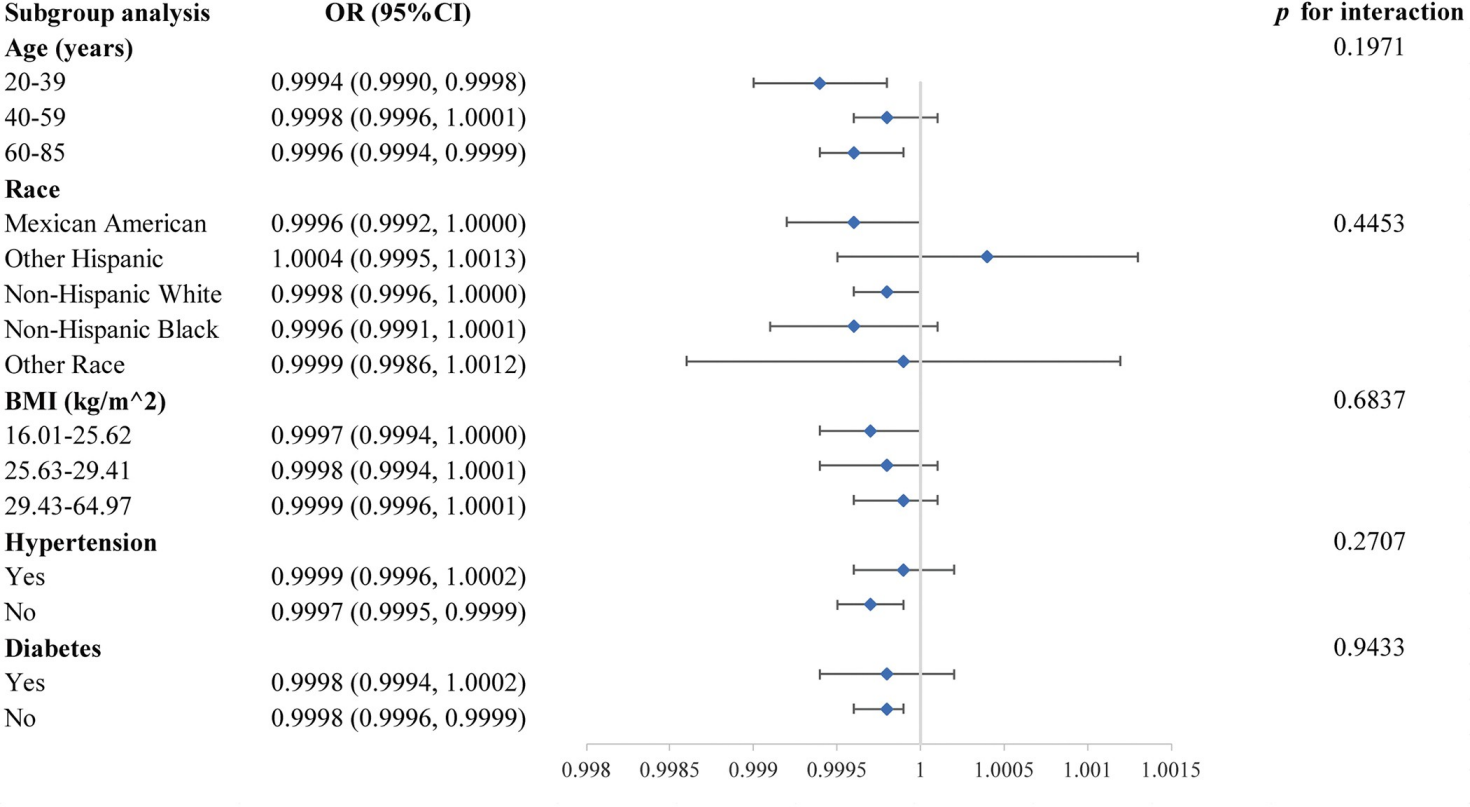
Subgroup analysis for the association between phosphorus consumption and erectile dysfunction.

A subgroup analysis of the relationship between phosphorus intake and erectile dysfunction is shown in Fig 4. However, no statistically significant *p* for interaction was found, showing that the correlation was independent of gender, age, BMI, hypertension, and diabetes (all *p* for interaction > 0.05). This suggests that the magnitude of the association is similar across populations.

**Fig 5:**
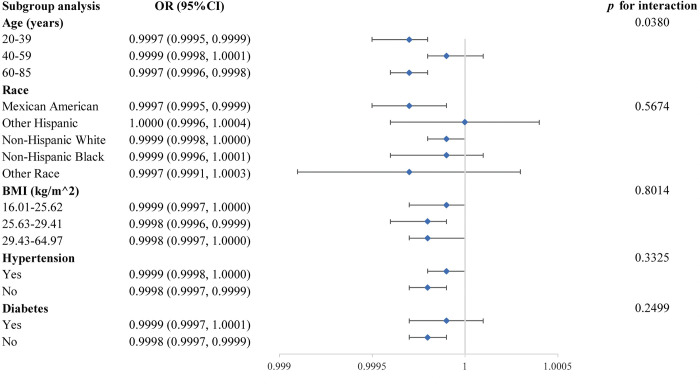
Subgroup analysis for the association between potassium consumption and erectile dysfunction.

We also did a subgroup study of the relationship between potassium intake and erectile dysfunction, which is shown in Fig 5. Similar to the subgroup analysis of the association between calcium intake and erectile dysfunction, we found a significant interaction for age (*p* for interaction<0.05). Furthermore, for each subgroup stratified by BMI and hypertension, there was a substantial negative correlation between potassium intake and erectile dysfunction. Furthermore, this significant inverse relationship was found in the Mexican American, non-Hispanic White, and no diabetes populations.

## 4. Discussion

The aim of this study was to investigate the relationships of calcium, phosphorus and potassium intake with erectile dysfunction. By including 3,356 subjects in a cross-sectional study, we found that calcium, phosphorus, and potassium were all negatively associated with erectile dysfunction. To our knowledge, this is the first study to examine the relationships of dietary calcium, phosphorus, and potassium with erectile dysfunction.

Penile erectile dysfunction is a prevalent problem among males. A multitude of integrated systems, such as vascular, neurological, endocrine, and patient psychological systems, collaborate to ensure the optimal erection of the penis. If any of these pathways are disrupted, it results in the initiation and progression of erectile dysfunction. Of all these pathways, the most common cause of erectile dysfunction is of vascular origin [14,15]. Multiple studies have established the importance of inflammation and oxidative stress in the initiation and advancement of erectile dysfunction [16]. Endothelial damage and dysfunction occur due to an imbalance between substances that relax the endothelium and those that contract it, when there is an increase in oxidative stress and inflammation levels [17]. Vascular endothelial cells release nitric oxide (NO) and acetylcholine (ACh) in healthy men during sexual excitement. Ach can also enhance the synthesis of NO. The conversion of guanosine triphosphate (GTP) to cyclic guanosine monophosphate (cGMP) by NO leads to cellular hyperpolarization and relaxation of smooth muscles. This promotes blood circulation to the penis, causing the corpus cavernosum to fill with blood and resulting in a significant increase in intracorporeal pressure, leading to an erection [14]. Endothelial dysfunction diminishes the sensitivity of the vascular endothelium to NO, hence impairing its normal physiological function. Moreover, it has an impact on the release of ACh, which in turn affects the synthesis of NO [14,18]. Moreover, endothelial cells have the ability to produce tumor necrosis factor-alpha (TNF-α) when exposed to inflammation and oxidative stress. TNF-α directly reduces the levels of nitric oxide (NO) while also stimulating the generation of reactive oxygen species (ROS) [14,19]. Based on prior investigations, erectile dysfunction is thought to be an early indicator of endothelial impairment. This also implies that its pathophysiological pathways may overlap with those involved in the development of certain cardiovascular diseases and that the presence of erectile dysfunction may raise the chance of developing cardiovascular diseases [20–23]. Therefore, a unified view of cardiovascular disease and erectile dysfunction helps us to better understand erectile dysfunction.

Calcium is abundant in dairy products, and our bodies rely on calcium intake to some extent [24]. Supplementing with calcium can lower the risk of atherosclerosis and stroke as well as overall mortality [25–27]. Moreover, an average daily consumption of 820 mg of calcium can reduce the risk of myocardial infarction by roughly 30%, according to a European prospective research study [28]. Although the exact mechanism is unknown, calcium has been shown to lower blood pressure in numerous studies. The mechanism may involve interactions between vitamin D and parathyroid hormone, which increase intracellular calcium levels and improve the responsiveness of vascular smooth muscle [29–31]. While previous studies have not investigated the relationship between dietary calcium consumption and erectile dysfunction, we hypothesized that consuming calcium reduces the occurrence of erectile dysfunction by protecting endothelial function, as indicated by the results of our study. Dietary calcium reduces vascular smooth muscle tone by down-regulating the activity of the renin-angiotensin system and improving sodium-potassium balance [32]. Additionally, calcium supplementation increases the concentration of calcium ions in the serum [33]. Calcium ions are essential for the production of vasoactive chemicals including NO and prostaglandins by vascular endothelial cells [34]. The reason for this could be the promotion of calcium-dependent mechanisms that activate the synthesis of endothelial nitric oxide synthase (eNOS), enhancing the bioavailability of NO in the vasculature, which can provide protection [35]. Austrian research has shown that calcium malnutrition impairs the activity of the extracellular calcium-sensitive receptor (CaSR). This impairment affects the K+ conductance channels in vascular endothelial cells, which in turn affects the regulation of vascular tone and arterial blood pressure and leads to vascular calcification [36]. However, it is important to remember that consuming too much calcium may have detrimental effects on cardiovascular health [37,38]. Therefore, maintaining a calcium intake of 500–1,000 mg per day may be an appropriate choice [9].

Our bodies require phosphorus as a vital nutrient, and the main sources of phosphorus intake are cereals, meat, and dairy products [39]. Despite numerous ongoing experiments, consensus has not yet been reached regarding the relationship between phosphorus and vascular endothelial function. Although most evidence indicates that phosphorus induces endothelial damage by increasing oxidative stress and reducing NO levels [40]. Conversely, some have suggested that there is no significant association between the amount of phosphorus consumed in the diet and the development of atherosclerosis [41]. In addition, McClure et al.’s comprehensive study did not find a consistent association between blood pressure and the total phosphorus content in meals [39]. It is important to highlight that the majority of prior research has generally concentrated on those with chronic kidney disease, people with hyperphosphatemia, or those who consume an excessive amount of phosphorus. Only a limited number of research have investigated the general population with a normal dietary pattern. These reasons could be considered valid explanations for the existence of the currently controversial findings. Hence, it is imperative to conduct prospective research encompassing diverse healthy populations and fundamental investigations at the molecular level to gain a deeper understanding of the correlation between phosphorus and vascular endothelial function. The connection between dietary phosphorus and erectile dysfunction has not received much investigation, and our findings add to the body of knowledge in this regard. Specifically, erectile dysfunction was 33% less common in participants in the highest tertile of phosphorus intake than in those in the lowest tertile. A plausible rationale is that elevated phosphorus consumption modifies the synthesis of sodium-phosphorus cotransporter proteins, resulting in a surplus of phosphorus expelled relative to intake. This negative phosphorus equilibrium could potentially be crucial in averting endothelial damage [42]. Another theory suggests that phosphorus exists in the blood as an anion and that accompanying cations, such as calcium and magnesium ions, may increase NO and slow oxidative stress, helping to prevent endothelial damage and atherosclerosis [39]. We discovered no nonlinear association between dietary phosphorus intake and erectile dysfunction, despite claims that hyperphosphatemia exacerbates vascular calcification and atherosclerosis. This may be because dietary phosphorus consumption has only a tiny impact on blood phosphorus concentration, which rises by 0.03 mg/dL with a 500 mg phosphorus intake. Additionally, serum phosphorus has a diurnal variation, making it possible for serum phosphorus concentrations measured at various times of the day to differ [43]. Further research is required to determine the significance of dietary phosphorus to erectile dysfunction.

For humans, potassium is an essential substance obtained from dietary sources, mainly from fruits and vegetables, to meet the body’s needs [44]. A systematic analysis by D’Elia et al. showed that increasing dietary potassium intake reduced the risk of stroke, coronary heart disease, and total cardiovascular disease [7]. Xie et al. showed a significant negative association between potassium intake and all-cause mortality based on a 10-year analysis of 143,050 individuals [45]. Our study also showed a negative correlation between dietary potassium and erectile dysfunction. Although the precise molecular processes by which dietary potassium improves erectile dysfunction are unknown, it is likely due to potassium’s antioxidant and vascular endothelial-improving properties. First, dietary potassium raises arterial compliance and enhances endothelial function [46]. Potassium may do this by causing the vascular endothelial cytoskeleton to relax, which then triggers the release of NO [47]. Furthermore, dietary potassium lowers endothelin-1 levels, which may reverse endothelial dysfunction brought on by a high-salt diet, contributing to this positive effect [48]. Another theory holds that potassium stimulates a number of calcium channels and sodium-calcium exchangers in addition to activating plasma membrane potassium channels and the sodium-potassium pump. These mechanisms then all contribute to causing endothelial cell hyperpolarization, which permits vasodilation [44]. Second, potassium promotes the relaxation of vascular smooth muscle by inhibiting the sympathetic nervous system by increasing norepinephrine uptake by nerve endings [49]. Furthermore, there is compelling evidence that potassium, via hyperpolarization-induced suppression of nicotinamide adenine dinucleotide phosphate (NADPH) oxidase activation, lowers plasma lipid peroxidation and endosomal lipid extracts and prevents the body from producing reactive oxygen species and insulin resistance [44]. When there is a lack of testosterone in the body, the cavernous smooth muscle is replaced by collagen fibers, which may lead to venous occlusion dysfunction and fibrosis in the body [50]. Furthermore, the advantageous impacts of dietary potassium on erectile dysfunction may stem from potassium’s capacity to enhance testosterone levels. Potassium has the potential to directly impact the production and release of testosterone by the cells in the testes, or it may have an indirect effect through the hypothalamic-thalamic axis [51]. Testosterone improves erectile function by affecting the nitriles responsible for vasodilation and phosphodiesterase-5, the enzyme responsible for cGMP degradation [52,53]. In conclusion, numerous robust studies have established that increasing dietary potassium intake considerably improves vascular endothelial function and testosterone levels and that these processes play an important role in the prevention of erectile dysfunction. It is important to keep in mind, nevertheless, that potassium consumption and blood pressure have a "U-shaped" relationship [54]. Therefore, it is advised that daily potassium consumption not exceed 3,800mg [55].

This study is subject to several limitations, notably its cross-sectional design, which hinders the ability to establish causal relationships. Erectile dysfunction in the subjects was assessed using questionnaires, which could potentially be influenced by recall bias. Furthermore, our study was exclusively carried out within the confines of the U.S. population, thereby raising uncertainty regarding the applicability of our results to people residing in other continents.

## 5. Conclusion

The primary outcome of this study was to formulate first ideas on the correlations of calcium, phosphorus, and potassium consumption with erectile dysfunction. Additional prospective studies are required to validate our findings.

## Supporting information

S1 Table Relationships of calcium, phosphorus, and potassium intake with erectile dysfunction when used as continuous variables. (DOC)

S1 Data Supporting information caption for file “original data.xls”: Original data used for this cross-sectional study. (XLS)
