# Supplementary material for: Dietary calcium, phosphorus, and potassium intake associated with erectile dysfunction in the National Health and Nutrition Examination Survey (NHANES) 2001 to 2004
Source: PLoS One. 2024 Feb 21;19(2):e0297129. doi: 10.1371/journal.pone.0297129 (PMC10880986; doi:10.1371/journal.pone.0297129)
Supplement: S1 Table — (DOC) [file pone.0297129.s001.doc]

**S1 Table.** **Relationships of calcium, phosphorus, and potassium intake with ED when used as continuous variables.**

| **Exposure** | **OR (95%CI), *P*-value** | | |
| --- | --- | --- | --- |
|  | **Model 1** | **Model 2** | **Model 3** |
| Calcium | 0.9994 (0.9992, 0.9995) <0.001 | 0.9997 (0.9995, 0.9999) 0.002 | 0.9998 (0.9996, 1.0000) 0.047 |
| Phosphorus | 0.9992 (0.9991, 0.9994) <0.001 | 0.9997 (0.9995, 0.9999) <0.001 | 0.9998 (0.9996, 0.9999) 0.005 |
| Potassium | 0.9997 (0.9997, 0.9998) <0.001 | 0.9998 (0.9997, 0.9999) <0.001 | 0.9998 (0.9998, 0.9999) <0.001 |

Model 1: crude mode.

Model 2: adjusted for age and race.

Model 3: adjusted for age, race, education level, marital status, income-to-poverty ratio (PIR), alcohol use, smoking, physical activity, hypertension, diabetes, body mass index (BMI), total cholesterol, and high-density lipoprotein (HDL).
